# Dynamic bandgap modulation in CsPbBr_3_ perovskite nanocrystals through reversible ammonia intercalation†

**DOI:** 10.1039/d4ra07759h

**Published:** 2025-02-04

**Authors:** Karayadi H. Fausia, Bijoy Nharangatt, Kavundath Muhsina, John P. Rappai, Raghu Chatanathodi, Deepthi Jose, Kulangara Sandeep

**Affiliations:** a Government Victoria College, Research Center under University of Calicut Palakkad 678001 India sandeepk@gvc.ac.in; b Department of Chemistry, MES Keveeyam College Valanchery Kerala 676552 India; c Department of Physics, National Institute of Technology Calicut Kerala 673601 India; d Government Arts and Science College Ollur Kerala India; e Department of Chemistry, Providence Women's College (Autonomous) Calicut 673009 India deepthijose@providencecollegecalicut.ac.in

## Abstract

Modulation of the electronic states of a semiconductor is an intriguing area of research because of its interesting applications. In general, physical methods are used to reversibly manipulate the bandgap of semiconductors. Herein, we have used a simple molecule, ammonia, and allowed it to intercalate inside the crystal lattice of CsPbBr_3_ perovskites to alter the band positions. The molecular intercalation of ammonia induces strain in the crystal structure of perovskite, which widens the bandgap. Ammonia intercalation results in fall-off of the visible absorption and emission of the CsPbBr_3_ perovskites and a new absorption emerges in the ultraviolet region. Interestingly, with time, the deintercalation takes place, as a result of the population in the antibonding orbitals formed due to the mixing of s orbital of the Pb and p orbital of N in the intercalated NH_3_. The deintercalation of gaseous ammonia results in the narrowing of the bandgap which results in the regaining of the visible absorption. Together with the density functional theory calculations, herein, we demonstrate the reversible bandgap modulation in CsPbBr_3_ perovskite nanocrystals. Aspects discussed here can give directions to develop newer methodologies to tune the band positions of semiconductors by the intercalation of the right molecules inside their crystal lattice.

## Abstract 2

Exposure to ammonia vapors on CsPbBr_3_-coated substrates results in the widening of a semiconductor's bandgap, which is reversible with time. The intercalation NH_3_-induced strain origins the bandgap widening.

## Graphical abstract

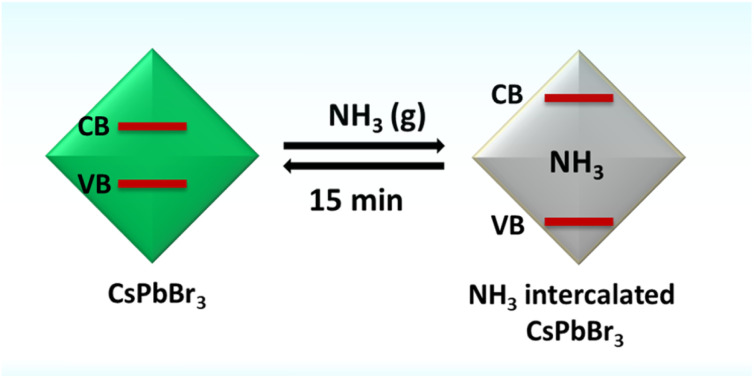

## Introduction

Extraordinary properties of lead halide perovskites, such as high photovoltaic efficiency, easier charge carrier extraction, large light absorption coefficient in broad wavelength regions, high emission yield, *etc.*, have made them promising candidates for the making of photovoltaic cells and optoelectronic devices like light emitting diodes (LED), display devices.^1–12^ Hitherto, lead halide perovskite is one of the light absorbing materials that expressed maximum efficiency for photovoltaic cells and LEDs.^13–19^ To improve the light-electrical energy conversion, the fabrication of solar cells has a huge role and one method among them is tandem solar cells.^20^ Here, semiconductors with different bandgaps are arranged in a sequential manner.^20^ Also, for the fabrication of some specific LEDs and display devices, the semiconductors emitting in different bandgaps are essential.^21^ Thus, the modulation of band positions in semiconductors by physical and chemical methods is always an interesting topic of research due to their profound applications in science and technology.^22–26^ In general, the bandgap of a semiconductor in the bulk state is fixed, however, at the nanoscale modulation is possible by varying the size and shape.^22,27,28^ Strain-induced bandgap tuning is another popular method employed for the tuning of the bandgap in semiconductors.^29^ Generally, mechanical stress is used to induce strain in semiconductors to alter the band positions in the bulk semiconductors and it is a tedious task.^29^ Intercalation of molecules/ions inside the semiconductor by chemical/physical methods can also induce crystal strain, which modifies the band positions.^29,30^ The crystal structure has a huge role in dictating the intercalation of molecules since the void size and the interaction between the atoms/ions stabilize the molecules inside a crystal lattice.^31^ However, the bandgap modulation by the intercalation of molecules is not well investigated in lead halide perovskites. The robust crystal structure of perovskites facilitates the manipulation of material properties which has a huge impact on the device applications.^32^ Strain in the perovskite can induce octahedral tilting which modifies the electronic band positions of semiconductors.^33^

The most popular method for bandgap engineering in lead halide perovskite is anion exchange.^34–38^ The general formula of lead halide perovskite is APbX_3_, where A is monovalent cation such as Cs^+^, HNH_3_^+^, CH_3_NH_3_^+^, and X is a halide ion.^35^ The size and electronegativity of the halide ions regulate the band positions of lead halide perovskites and the bandgap of the semiconductor decreases in the order Cl^−^, Br^−^ and I^−^.^35^ By this method, the perovskites emission in the entire visible region can be modulated, post-synthetically, in an easier manner.^35^ However, new methodologies have to be developed in order to manipulate the band positions of perovskite semiconductors.^39^ Herein, we are planning to use the intercalation of small molecules in perovskite crystal structure to modulate the bandgap. Most of the chemical intercalations are irreversible in nature and thus the modulation of the physical properties are difficult. A reversible intercalation can be a better choice in order to modulate the band positions in a semiconductor. In the present work, we are using ammonia in the gaseous state to manipulate the properties of all inorganic CsPbBr_3_ perovskites nanocrystals.

## Results and discussions

All inorganic CsPbBr_3_ perovskite is known for its excellent photophysical properties and stability.^40–42^ Herein, we have synthesized CsPbBr_3_ perovskite nanocrystals by following earlier known protocols.^40,43^ Detailed information regarding the preparation/purification of the all inorganic CsPbBr_3_ nanocrystals can be found in the Experimental section. In short, as the initial step, Cs_2_CO_3_ is allowed to react with oleic acid to form a clear solution of cesium oleate at 120 °C. In the subsequent step, the cesium oleate is rapidly injected into the Pb–oleylamine complex, to acquire CsPbBr_3_ perovskite nanocrystals. The Pb–oleylamine complex was prepared by the reaction of lead bromide with oleylamine and oleic acid in octadecene. The obtained perovskite nanocrystals are purified by over-and-over precipitation and washing with spectroscopic-grade acetone. Further, the nanocrystals are dissolved in good quality chloroform (spectroscopic grade) and stored in dark. Chloroform solvent is known to induce photochemical anion exchange with perovskite nanocrystal and it is necessary to store the dispersion in absence of light.^44^

**Fig. 1:**
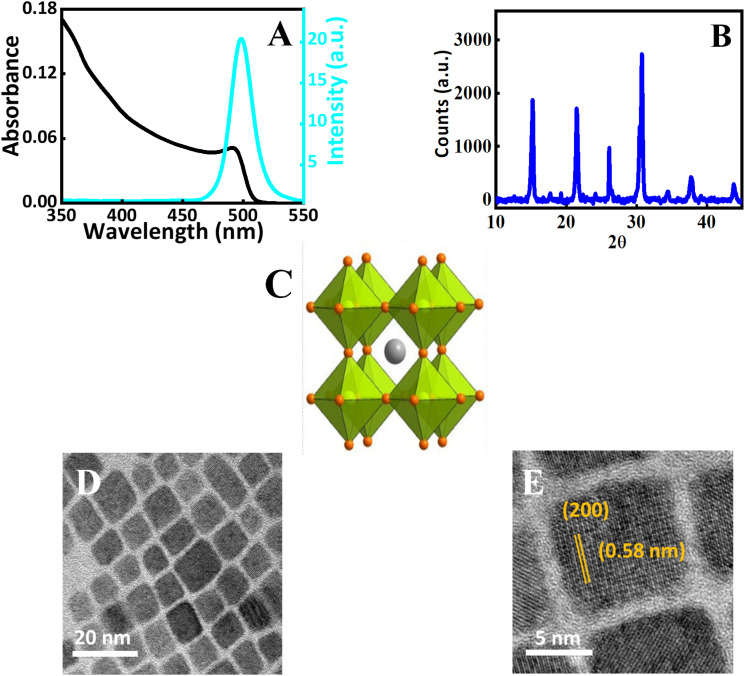
Characterization of perovskite nanocrystals by spectroscopic, X-ray diffraction techniques, and electron microscopy. (A) UV-vis absorption spectrum (black trace) of the CsPbBr_3_ nanocrystals dispersed in chloroform and emission spectrum (cyan trace). The emission spectrum is monitored by exciting at 355 nm by keeping the slit widths at excitation and emission chambers at 1 nm. (B) XRD pattern, (C) Schematic representation of the crystal structure of CsPbBr_3_ perovskite nanocrystals, (D) low-resolution TEM (E) high-magnification TEM image of the perovskite nanomaterials.

Detailed characterization of the purified CsPbBr_3_ perovskite nanocrystals is conducted using spectroscopic techniques and electron microscopy (Fig. 1A). The quantum confinement effect of the CsPbBr_3_ nanocrystals is evident by the appearance of an excitonic peak in the absorption spectrum (Fig. 1A). For the present perovskite nanocrystals showed it is centered around 498 nm. Additionally, emission spectroscopy is employed to characterize the perovskite nanocrystals and it showed a symmetrical Gaussian-like emission spectrum with a photoluminescence maximum at 505 nm. The emission spectrum exhibited a full width at half maximum (FWHM) of 19 nm which indicates the present nanocrystals are mono-disperse with minimal surface defects.^45^ Also, we have quantified the emission yield of the nanocrystals used in this study, by a relative method were fluorescein dye in 0.1 molar sodium hydroxide (quantum yield – 0.925), is used as the reference dye, and the emission quantum yield of the nanocrystals is found to be 0.49. Details are provided in the ESI.†

Further X-ray diffraction analysis is carried out to understand the crystalline properties. Diffraction patterns at 2*θ* values 15.7°, and 30.9° correspond to the (100) and (200) lattice planes of the CsPbBr_3_ perovskite nanocrystals (Fig. 1B). The diffraction peaks obtained at 2*θ* near 22° and the above mentioned planes indicate the crystal structure of the present nanocrystal is orthorhombic perovskites.^46^ The diffraction patterns are in accordance with the Joint Committee for Powder X-ray Diffraction Standards (JCPDS) and earlier known reports.^44,46^ Using the Debye–Scherrer equation, the grain size of the nanocrystals were calculated and the details are given in ESI. The schematic crystal structure of the nanocrystals is presented in Fig. 1C. Transmission electron microscopy (TEM) is used to analyse the nanocrystals and the results are presented in Fig. 1D and E. The low-resolution TEM image, further confirms that the perovskite nanocrystals are monodisperse (Fig. 1D). The high-magnification TEM image agrees that the nanocrystals are crystalline. Further, the *d*-spacing of the perovskite nanocrystals is estimated from these techniques using Gatton Digital micrograph software. The obtained *d*-spacing value of 0.58 nm corresponds to the (200) plane of the perovskite nanocrystal. The details are presented in ESI.†

Further, we have investigated the role of intercalation of small molecules in the crystal structure of lead halide perovskites. In an earlier study, we showed the intercalation of water molecules in vapor state is possible inside the perovskite lattice.^47^ However, this results in the decomposition of the semiconductor.^47^ At the same time, a few molecules like hydrogen sulfide are known to adsorb on the surface of the crystal instead of intercalation.^47^ In order to induce strain, herein we have chosen ammonia as the intercalating molecule. The solution state experiments with vapor ammonia are hard to perform and thus we have performed the experiments after immobilizing CsPbBr_3_ perovskite nanocrystals in a solid support.^48^ Also, the experiments with the solid support have more applications in science and engineering. In this work, we have immobilized CsPbBr_3_ nanocrystals in a paper/glass substrate. The CsPbBr_3_ coated Whatman 40 paper substrate is prepared by dip coating method and the glass substrate by spin coating. The details are given in the Experimental section. The vapor pressure of the normally available (30%) ammonia solution is 0.4537 kPa. The details of the calculation of vapor pressure using Raoult's law is available in ESI.†

**Fig. 2:**
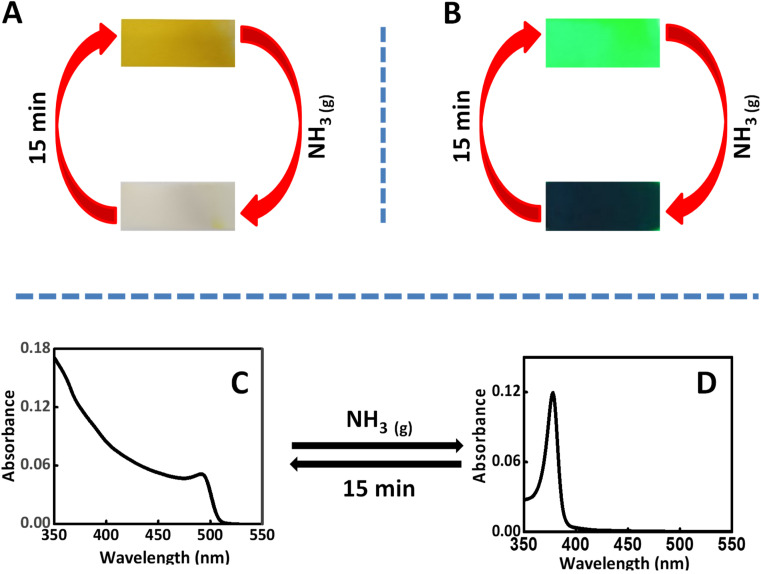
The influence of ammonia intercalation on the properties of CsPbBr_3_ perovskite Nanocrystals. (A) Photographs showing the reversible intercalation of ammonia vapors in the CsPbBr_3_ crystals coated on a paper substrate viewed under normal light and (B) ultraviolet light. UV-visible absorption spectra of CsPbBr_3_ nanocrystals before (C) and after (D) ammonia intercalation.

The intercalation of ammonia vapors inside the perovskite nanocrystal is achieved by showing the CsPbBr_3_ coated paper/glass substrate over the 30% of aqueous ammonia solution, for 30 s. Interestingly, the initial yellow color of the perovskite substrates disappeared in the presence of ammonia vapors. The details are presented in Fig. 2A. Further, the same experiment is carried out under ultra-violet light (*λ* = 356 nm). The initial green emission completely disappeared after the treatment of ammonia vapors (Fig. 2B). The disappearance of visible absorption/emission can be attributed to the alteration in the bandgap of CsPbBr_3_ perovskite nanocrystals. The ammonia Intercalation can induce strain in the semiconductor crystals and which widens the bandgap of CsPbBr_3_ perovskites. As a result, the visible absorption/emission of the semiconductor disappears. Interestingly, with time, the deintercalation of ammonia takes place which results in the restoration of initial absorption/emission. In our case, with 15 min the emission/absorption of perovskite crystals is regained. Further, we have repeated the experiments for 5 cycles and found the process is reversible (Fig. S1, ESI†). However, due to the presence of moisture in the ammonia solution, the intensity of the retrieved emission is found to decrease with the increase in the number of cycles. These experiments demonstrate that the initial disappearance of the yellow color of the perovskite nanocrystals, in the presence of ammonia, is due to the band widening.

Further, in order to confirm the above observations, UV-visible absorption studies of the perovskite-coated glass substrates, in the presence and absence of ammonia are conducted using diffused reflectance method. The initial absorption (Fig. 2C) showed the all-characteristic feature of CsPbBr_3_ perovskite nanocrystals. Interestingly, in the presence of ammonia, the visible absorption quenched and a new absorption peak emerged around 376 nm (Fig. 2D). Here also, the process is perfectly reversible with time. From the Fig. 2D, it is clear that the ammonia intercalated CsPbBr_3_ crystals don't have any absorption in the visible region. In methyl ammonium perovskite, the structural transformation in the presence of ammonia is well investigated by Ptasinska and co-workers.^49^ Ammonia converts CH_3_NH_3_PbI_3_ to NH_4_PbI_3_ and which is reversible only in the presence of CH_3_NH_2_. In our case, it's reversible with time without the addition of any external reagents.^49^ In another, report, this property of lead halide perovskite is used to sense ammonia.^50–54^ Amine sensing using lead halide perovskite is also known in the literature.^55,56^ Further, the powder XRD experiments are carried out in the presence and absence of ammonia (Fig. S2, ESI†) and the TEM images as Fig. S3.† In concordance with the earlier reports, we didn't observe changes in the diffraction patterns.^49^ This experiment confirms that the basic crystal structure is not altered after the intercalation of ammonia into CsPbBr_3_ perovskite structure. Also, the TEM images taken before and after intercalation of ammonia vapors are shows that process is reversible. Further, we have carried out the thermogravimetric analysis and the results are presented in Fig. S4.† The results clearly indicates the ammonia is deintercalated in the initial temperature.

In order to understand the mechanistic features of the reversible ammonia intercalation in CsPbBr_3_ perovskite nanocrystals, we employed Density functional theory (DFT) calculations using the Vienna *Ab initio* Simulation Package (VASP).^57,58^ The electron–ion interaction is represented through the Projector Augmented Wave (PAW) method, as known in the earlier reports.^59^ The Generalized Gradient Approximation (GGA) based Perdew–Burke–Ernzerhof (PBE) functional is used to approximate the exchange–correlation.^60^ Also, the dispersion corrections as given by Grimme are included in these calculations. The solutions of Kohn–Sham equations were expanded in a plane-wave basis set with a kinetic energy cut-off of 500 eV. To investigate intercalation interactions between CsPbBr_3_ and NH_3_, we considered an orthorhombic unit cell of CsPbBr_3_, comprising four CsPbBr_3_ formula units, maintaining its lattice cell parameters consistent with the experimental values and earlier known methods.^61^ The energy needed for intercalation of NH_3_ (*E*_int_) into CsPbBr_3_ nanocrystal was calculated using the equation

1 *E*_int_ = *E*_CsPbBr_3_·NH_3__ − (*E*_CsPbBr_3__ + *E*_NH_3__)

where *E*_CsPbBr_3_·NH_3__, E_CsPbBr_3__, and *E*_NH_3__ are the total energies of bulk CsPbBr_3_ intercalated with NH_3_, bulk orthorhombic CsPbBr_3_ unit cell, and an isolated NH_3_ molecule, respectively. We further calculated the distortion energy of CsPbBr_3_ due to NH_3_ intercalation as the difference in energy between the CsPbBr_3_ unit cell after intercalation (without NH_3_) and the pristine CsPbBr_3_ unit cell.

**Fig. 3:**
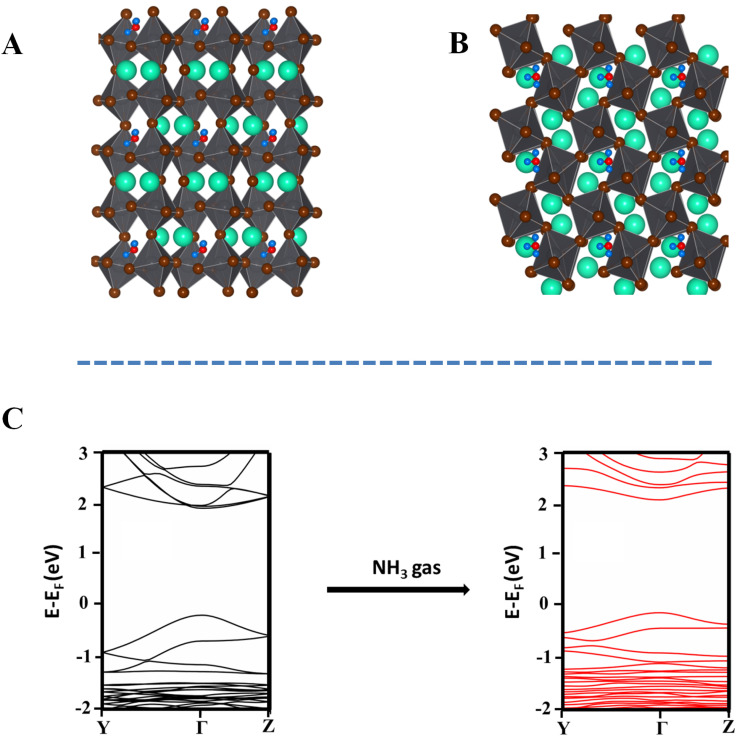
The optimized geometry of NH_3_ intercalated CsPbBr_3_ perovskite (A) side view and (B) top view. The band structure plot for (C) pristine CsPbBr_3_ before and after intercalation of ammonia.

The optimized geometry of NH_3_ intercalated orthorhombic CsPbBr_3_ perovskite is shown in Fig. 3. The computed intercalation energy of −0.91 eV for a single NH_3_ molecule with orthorhombic CsPbBr_3_ indicates a strong attractive interaction between NH_3_ molecule and CsPbBr_3_ nanocrystal. In addition, the intercalation of NH_3_ molecule induced octahedral distortion in orthorhombic CsPbBr_3_ resulting in a maximum Pb–Br–Pb angle change of 2.15%, the details of which are given in the ESI (Fig. S5, ESI).†

The intercalation of a single NH_3_ molecule resulted in a distortion energy of −0.38 eV. It was observed that, with an increase in the ammonia concentration, the distortion energy became positive, dominating the intercalation energy and hence, the major decisive factor in determining the stability of the intercalated complex. Consequently, we confined the electronic structure calculations to the intercalation with a single ammonia molecule. Furthermore, the band structure NH_3_ intercalated CsPbBr_3_ was calculated to investigate the effect of ammonia intercalation on the electronic structure of CsPbBr_3_Fig. 3C. The bandgap of pristine CsPbBr_3_ and NH_3_ intercalated CsPbBr_3_ was calculated to be 2.09 eV and 2.2 eV respectively using GGA functional. The experimental results also show that the bandgap of the semiconductor is widened upon the intercalation of ammonia inside the CsPbBr_3_ perovskite nanocrystals.

**Fig. 4:**
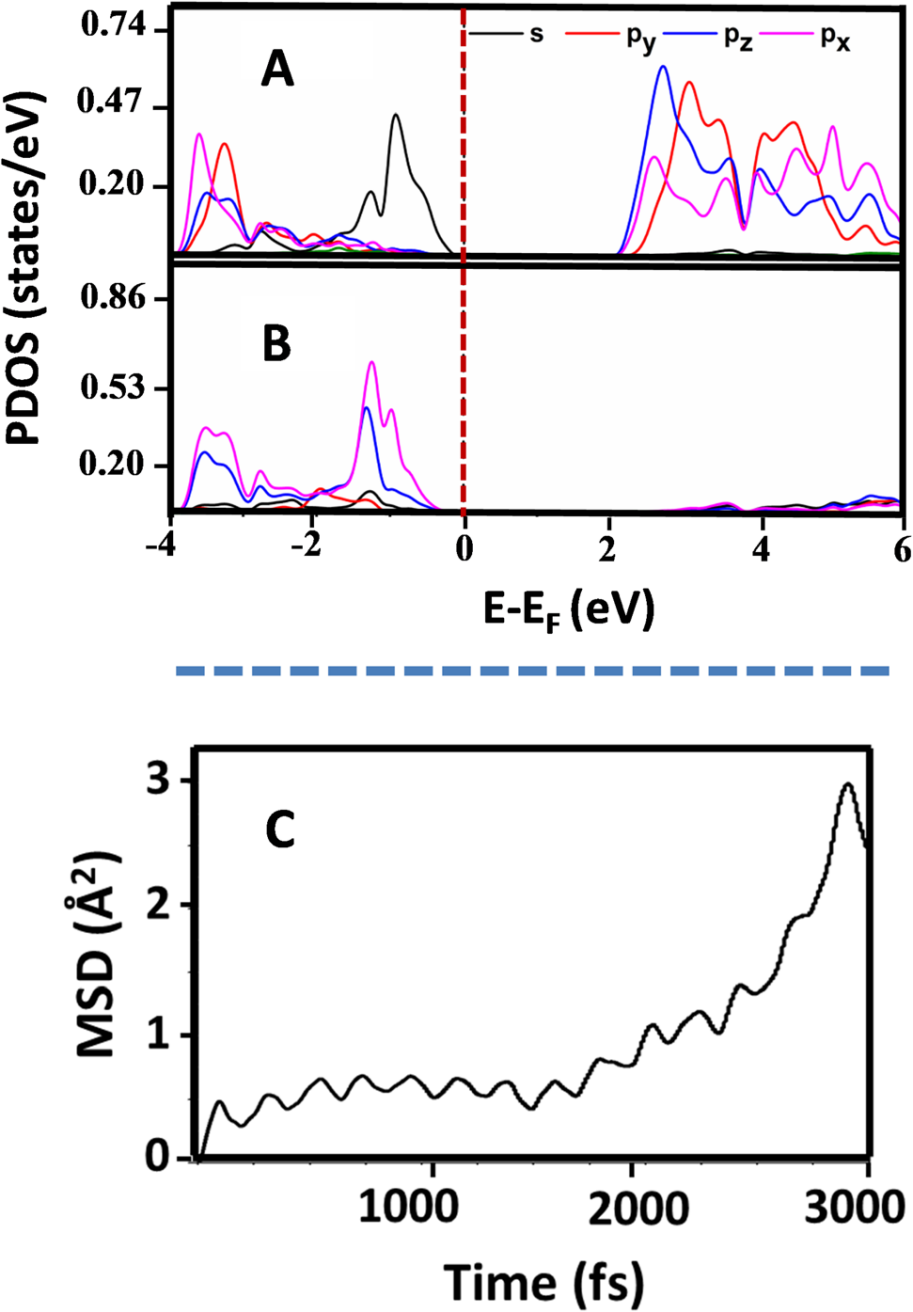
The PDOS plot for (A) Pb atom (B) N atom. (C) The mean square displacement plot for intercalated NH_3_ molecule in CsPbBr_3_ unit cell.

Further, in order to understand the mechanism of reversible intercalation, we calculated and plotted the Projected Density of States (PDOS) plot for Pb and N atoms of NH_3_ intercalated CsPbBr_3_ as shown in Fig. 4 A, B. The intercalation of the ammonia molecule results in the mixing of p orbitals of the N atom and s orbitals of the Pb atom, generating more antibonding states in Pb s states (Fig. S6, ESI†). The instability due to the formation of more antibonding states in Pb s states during the intercalation together with entropic favourability during the release of NH_3_ molecule can be attributed to the reversibility of NH_3_ intercalation in CsPbBr_3_. To further understand the stability and reversibility of NH_3_ intercalated perovskite nanocrystal, *ab initio* molecular-dynamics (AIMD) simulations were performed with a 3000 fs time step at room temperature employing the Nosé-Hoover thermostat for the intercalated system. The mean square displacement of intercalated NH_3_ molecule in the CsPbBr_3_ unit cell calculated and plotted over time (Fig. 4C) indicates a gradual release of the NH_3_ molecules from the CsPbBr_3_ unit cell in accordance with the experimental observations.

The manipulation of electronic structures of the semiconductors by various methodologies has a huge impact on the device fabrication and their performance. For this purpose, most of the physical methods are hard to execute in simple laboratories and the chemical methods are irreversible in nature. The present investigation shows a reversible modulation of electronic structure in CsPbBr_3_ perovskite nanocrystals by the intercalation of small molecules. A proper understanding of the mechanism of intercalation and the energy associated with the distortion can be used to modulate the bandgap of the semiconductors. Manipulating the orbital interactions after the intercalation can dictate whether the process is reversible or not. Furthermore, the intercalation-induced strain can be used to manipulate the electronic states and magnetic properties.

## Conclusion

In summary, the effect of the intercalation of gaseous ammonia on the widening of bandgap in the CsPbBr_3_ perovskite nanocrystal is investigated. In the presence of vapor ammonia, the absorption in the visible region of perovskite nanocrystals got diminished. With time, visible absorption/emission regained as a result of the deintercalation of NH_3_ from perovskite nanocrystals. The strain formed as a result of the intercalation of ammonia vapors, causes an octahedral distortion in perovskite crystals which modifies the electronic structure of semiconductors. The formation of new antibonding states after the intercalation of ammonia in perovskite crystals, due to the mixing of Pb s orbital and p orbital of N from the ammonia leads to the deintercalation of the molecule, with time, at room temperature. The experimental observation and theoretical calculations clearly show the feasibility of reversible intercalation of ammonia in the CsPbBr_3_ perovskite nanocrystal. The insights given in this paper can give direction to modulate the material properties by simple chemistry. Also, intercalating the right molecule inside the semiconductor crystal structure can facilitate the precise tuning of band positions which has a huge impact on the fabrication of semiconductor devices.

## Experimental section

### Synthesis of CsPbBr_3_ perovskite nanocrystals

Cesium lead halide perovskite nanocrystals are prepared using a standard reported method with slight modification.^40^ This was achieved by a two-step process. In which, Cs-oleate was synthesized by adding cesium carbonate and oleic acid in the molar ratio of 1 : 2. This reaction was carried out in a three-neck RB flask under an argon atmosphere. The mixture was heated to a temperature of 120 °C to yield a clear Cs-oleate solution that is optically transparent. Lead bromide (0.19 mmol, 69.73 g) was taken in another round bottom flask and combined with octadecene (solvent, 4 mL), capping agents oleyl amine (1.14 mmol, 1.6 mL) and oleic acid (0.5 mmol, 0.15 mL) in an argon/nitrogen atmosphere and as raise the temperature up to 150 °C, to obtained lead–oleyl amine complex. Cs-oleate (0.046 mmol) is promptly injected into lead–oleyl amine complex at a temperature of 170 °C under inert atmosphere. This yields lead halide perovskite nanocrystals with a yellow color. The obtained perovskites are purified by over and over precipitation and washing by acetone (spectroscopic grade) and followed by normal centrifugation at 4000 rpm.

### Fabrication of CsPbBr_3_ perovskite coated paper/glass substrate

The CsPbBr_3_ nanocrystals dispersed in chloroform were coated on a Whatman 40 filter paper using the dip coating method under inert conditions. Following this, the paper is allowed to dry in a nitrogen atmosphere and the process is repeated a minimum up to 5 times to get a CsPbBr_3_ nanocrystals coated Whatman 40 filter paper. Being volatile, chloroform quickly evaporates in the nitrogen environment. The CsPbBr_3_ perovskite nanocrystals were spin-coated onto a cleaned glass substrate followed by drying under an inert atmosphere.

## Data availability

All data underlying the results are available as part of the article, and no additional source data are required.

## Conflicts of interest

The authors declare no competing financial interest.

## Supplementary Material

RA-015-D4RA07759H-s001
