# Supplementary material for: Dynamic bandgap modulation in CsPbBr3 perovskite nanocrystals through reversible ammonia intercalation
Source: RSC Adv. 2025 Feb 4;15(5):3562–9. doi: 10.1039/d4ra07759h (PMC11792498; doi:10.1039/d4ra07759h)
Supplement: RA-015-D4RA07759H-s001 [file RA-015-D4RA07759H-s001.pdf]

Supplementary Information (SI) for RSC Advances.
This journal is © The Royal Society of Chemistry 2025

Electronic Supporting Information:

Dynamic Bandgap Modulation in $CsPbBr_3$ Perovskite Nanocrystals through Reversible Ammonia Intercalation

Karayadi H. Fausia,[a,b] Bijoy Nharangatt,[c] Kavundath Muhsina,[a] John P. Rappai,[a,d] Raghu Chatanathodi,[c] Deepthi Jose,[e,*] Kulangara Sandeep [a,*]

[a] Government Victoria College, Research Center under University of Calicut, Palakkad 678001, India.

[b] Department of Chemistry, MES Keveeyam College Valanchery, Kerala, 676552 India.

[c] Department of Physics, National Institute of Technology, Calicut, Kerala, 673601 India.

[d] Government Arts and Science College, Ollur, Kerala, India.

[e] Department of Chemistry, Providence Women's College, Calicut, 673009 India

*E-mail: deepthijose@providencecollegecalicut.ac.in (D.J.); sandeepk@gvc.ac.in (K.S.)

Table of Contents	Page No.
1 Materials and methods	**S3**
2 Emission quantum yield measurements	**S3**
3 Estimation of grain size of $CsPbBr_3$ from XRD	**S4**
4 Calculation of the vapor pressure of ammonia solution	**S4**
5 Ammonia treatment: Reversibility of the substrates	**S5**
6 Characterization of $CsPbBr_3$ before and after ammonia treatment	**S5**
7 Octahedral tilting in ammonia treated samples	**S7**
8 PDOS plot	**S7**

1. Materials and methods

Chemicals: Cesium carbonate (Cs_2CO_3, 99.99%), lead bromide ($PbBr_2$), oleylamine (90%), and octadecene (98%) are purchased from Sigma-Aldrich. Oleic acid (98%) is obtained from Alfa-Aesar. Chloroform (Spectroscopic grade), acetone and 30% of ammonia solution are purchased from Merck chemicals. All chemicals and solvents are used without additional purification.

Instrumental methods: The electronic absorption spectra is recorded using a Shimadzu double beam spectrophotometer (Model UV-3600, UV-Vis-NIR), which is connected two sources (hydrogen – deuterium discharge tube and tungsten lamp) with three detectors (PMT, In GaAs, and PbS). Photoluminscence spectrum is recorded using JobinYvon SPEX- Fluorolog 3 from Horiba, where a 450 W Xenon arc lamp is used as the source for exciting samples. Also, in both the emission and excitation chambers, double monochromators are used. Side-on R928P photomultiplier tube (180-850 nm) is used to detect the emission spectrum. X-ray diffraction (XRD) analysis is carried out using a PANalytical X'Pert Pro diffractometer it has has a Ni-filtered Cu-Kα line with a wavelength of 0.1540598 nm (2θ adjustable from 10° to 50° in 0.02^o increments). For TEM, the sample is drop-casted onto a carbon-coated Cu grid and the solvent is allowed to evaporate. Specimens are imaged on an FEI TECNAI 30 G^2HRTEM instrument. Software namely Gatan digital micrograph is used to obtain the d-spacing value in Z- direction.

2. Emission quantum yield measurements

Photoluminscence quantum yield of the $CsPbBr_3$ perovskite nanocrystals are measured by following a relative method, in which fluorescein dye dissolved in 0.1 M NaOH (Φ_{ST}: 0.9) is used as standard. The equation S1 is used for the estimation of photoluminscence quantum yield.

$$\Phi_x = \Phi_{ST}\,(f_x/\,f_{ST})\,(a_{ST}/a_X)\,(n_x^2/\,n_{ST}^2) \qquad \textbf{(S1)}$$

f_x = Emission area of the sample

f_{ST} = Emission area of the standard reference

n_x= Refractive index of the solvent of the perovskite sample

n_{ST}= Refractive index of the solvent of the reference standard

a_{ST}= Absorbance of the reference standard at the excitation wavelength

a_X = Absorbance of the sample at excitation wavelength

Φ_{ST}= Quantum yield of the standard.

3. Estimation of grain size of $CsPbBr_3$ from XRD

As per Debye-Scherrer equation, we have calculated the grain size of $CsPbBr_3$ perovskite nanocrystals.

$$L = \frac{0.9\,\lambda}{\beta \cos\theta} \qquad \textbf{(S2)}$$

where L is the grain size of crystals, β denotes full width at half maximum (FWHM) in radians of the diffraction peaks, θ is the Bragg's diffraction angle and λ is the wavelength for the Kα component of the employed copper radiation (wavelength: 0.1540598 nm).

d-spacing calculation was done based on Bragg's equation.

$$n\,\lambda = 2d \sin\theta \qquad \textbf{(S3)}$$

n can be 0,1,2,-------, θ is the Bragg's diffraction angle , λ is the wavelength for the Kα component of the employed copper radiation (wavelength: 0.1540598 nm) and d is the interplanar distance.

Calculation of vaour pressure of ammonia solution

4. **Calculation of the vapor pressure of ammonia solution**

According to Raoult's law equation, we have calculated 30% of vapor pressure of ammonia solution (commercially).

$$Pa = P^{o}a\ Xa \qquad \textbf{(S4)}$$

Where $P^{o}a$ at 30° C for pure ammonia is 1166 kPa.

5. Ammonia treatment: Reversibility of the substrates

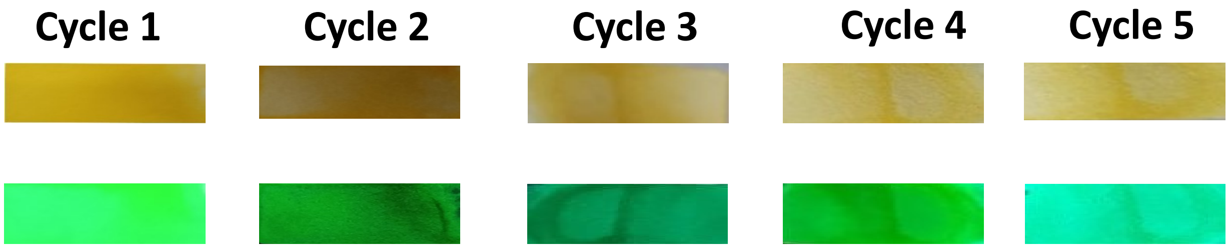

Fig. S1: Paper substrate viewed under visible and UV (356 nm) light under each cycle.

6. Characterization of $CsPbBr_3$ before and after ammonia treatment

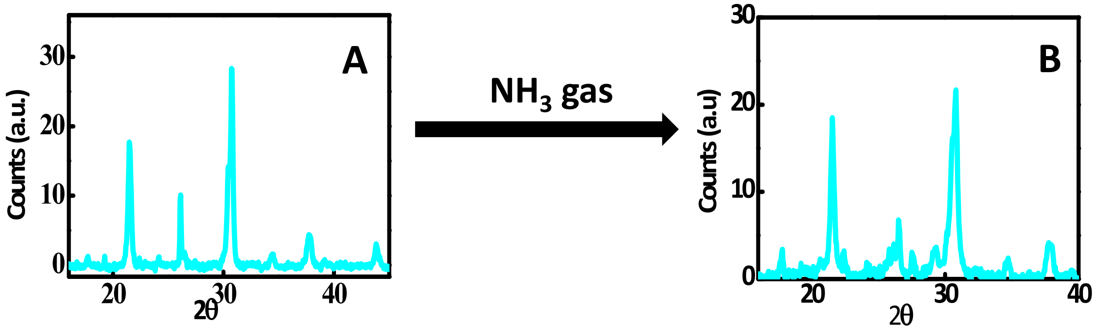

Fig. S2: XRD patterns of $CsPbBr_3$ perovskite nanocrystals before (A) and after (B) gaseous ammonia exposure.

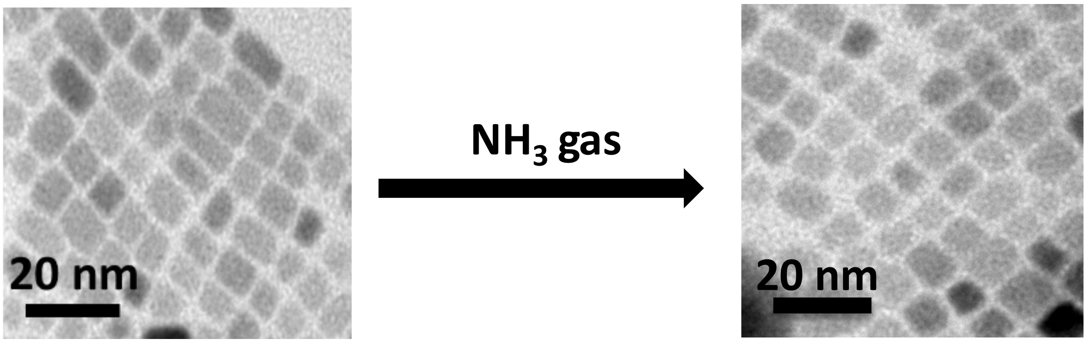

Fig. S3: TEM images of $CsPbBr_3$ perovskite nanocrystals before (A) and after (B) gaseous ammonia exposure.

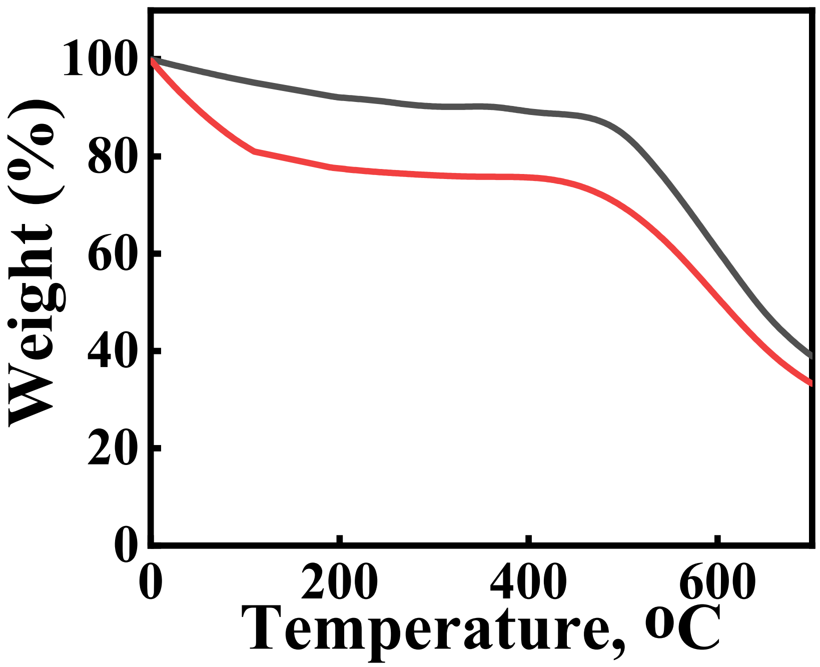

Fig. S4: TGA analysis of $CsPbBr_3$ perovskite nanocrystals before (black trace) and after (red trace) gaseous ammonia exposure.

7. Octahedral tilting in ammonia treated samples

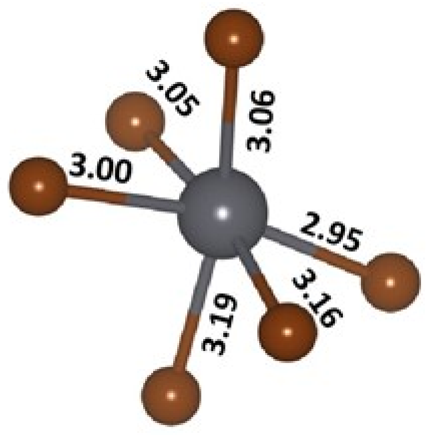

Fig. S5: Variations in Pb-Br Bond length of $CsPbBr_3$ after NH_3 intercalation.

8. PDOS Plot

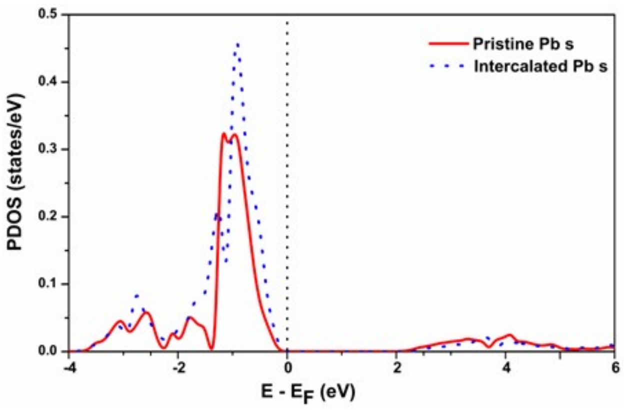

Fig. S6: The PDOS plot for the s orbital Pb atoms of before and after the intercalation of NH_3.
